# Development and Validation of the Steatotic Liver Disease Lab Data Index: A Refined Diagnostic Tool

**DOI:** 10.1155/cjgh/7366206

**Published:** 2026-06-30

**Authors:** Weiting Liaw, Pei-Chien Tsai, Ming-Lung Yu, I-Jung Feng

**Affiliations:** ^1^ Institute of Precision Medicine, National Sun Yat-sen University, Kaohsiung, Taiwan, nsysu.edu.tw; ^2^ Department of Internal Medicine, Hepatobiliary Division, Kaohsiung Medical University Hospital, Kaohsiung, Taiwan, kmuh.org.tw; ^3^ Center of Hepatitis Research, College of Medicine, Kaohsiung Medical University, Kaohsiung, Taiwan, kmu.edu.tw; ^4^ Center of Metabolic Disorders and Obesity, College of Medicine, Kaohsiung Medical University, Kaohsiung, Taiwan, kmu.edu.tw; ^5^ Center of Excellence for Metabolic Associated Fatty Liver Disease, National Sun Yat-sen University, Kaohsiung, Taiwan, nsysu.edu.tw; ^6^ Department of Oral Hygiene, Kaohsiung Medical University, Kaohsiung, Taiwan, kmu.edu.tw

**Keywords:** metabolic dysfunction-associated steatotic liver disease (MASLD), NHANES, noninvasive markers, sex differences, steatotic liver disease

## Abstract

**Aims:**

Steatotic liver disease (SLD) is highly prevalent, yet large‐scale identification remains challenging because imaging is not always available and existing noninvasive indices perform heterogeneously across subgroups. We developed and validated sex‐ and age‐stratified laboratory‐based indices for simplified SLD identification.

**Methods:**

We analyzed 2085 adults from NHANES III (1988–1994). An exposure‐wide association study (EWAS) of 119 clinical features informed sex‐ and age‐stratified logistic models to derive two SLD lab data indices (SLDLD1‐2) and simplified categorical versions (SSLDLD1‐2). Discrimination (AUROC) was compared with FLI, HSI, NAFLD‐LFS, and US‐FLI using paired DeLong tests. Bootstrap internal validation, comparison with a pooled nonstratified model, and decision curve analysis were additionally performed. External validation was performed for SSLDLD1 in NHANES 2017‐2018 and the Taiwan MJ Health database. SLDLD2/SSLDLD2 were not externally validated because C‐peptide was unavailable in both datasets.

**Results:**

Central adiposity and insulin‐resistance markers were key predictors, whereas β‐carotene showed an inverse association and contributed to discrimination, particularly in men. Across strata, SLDLD1 achieved AUROCs of 0.71–0.79 in derivation. The stratified framework outperformed a pooled nonstratified model in the overall cohort, in men, and in women aged < 55 years. SLDLD1 also outperformed FLI and HSI across strata and showed superior or comparable performance versus NAFLD‐LFS and US‐FLI, with stratum‐dependent differences. External validation of SSLDLD1 yielded AUROCs of 0.71–0.80 across datasets. Feature‐reduction analysis and an MJ‐variables‐only implementation of SSLDLD1 showed only small absolute AUROC changes overall, supporting simplified implementation when nonroutine biomarkers such as *β*‐carotene and insulin are unavailable, although fixed NHANES III‐derived thresholds were not fully transferable to the MJ cohort.

**Conclusion:**

The stratified SLDLD framework provides a practical toolbox for SLD identification, supporting high‐throughput research and implementation in settings where advanced imaging is not readily available. SSLDLD1 showed acceptable external discrimination across U.S. and Taiwanese datasets and may be more feasible for settings without nonroutine biomarkers, although local recalibration and validation of decision thresholds are needed before implementation across populations.

**Abbreviations:** AASLD, American Association for the Study of Liver Diseases; AIC, Akaike information criterion; ALP, alkaline phosphatase; ALT, alanine aminotransferase; AST, aspartate aminotransferase; AUROC, area under the receiver‐operating. characteristic curve; BMI, body mass index; CAP, controlled attenuation parameter; CI, confidence interval; CMRF, cardiometabolic risk factor; DM, diabetes mellitus; EWAS, exposure‐wide association study; FLI, fatty liver index; FSI, Framingham steatosis index; FPG, fasting plasma glucose; GGT, gamma‐glutamyl transferase; HbA1c, glycated hemoglobin; HCC, hepatocellular carcinoma; HDL, high‐density lipoprotein; HOMA‐IR, homeostasis model assessment of insulin resistance; HSI, hepatic steatosis index; IR, insulin resistance; LDL, low‐density lipoprotein; MCHC, mean corpuscular hemoglobin concentration; MJ, MJ Health (Taiwan); NAFLD, nonalcoholic fatty liver disease; NAFLD‐LFS, nonalcoholic fatty liver disease liver fat score; NHANES, National Health and Nutrition Examination Survey; NPV, negative predictive value; PPV, positive predictive value; SLD, steatotic liver disease; SLDLD, steatotic liver disease lab data; SLDLD1/SLDLD2, steatotic liver disease lab data indices 1/2; SSLDLD, simplified steatotic liver disease lab data; SSLDLD1/SSLDLD2, simplified steatotic liver disease lab data indices 1/2; TG, triglyceride; US‐FLI, US fatty liver index; VCTE, vibration‐controlled transient elastography; WC, waist circumference; WHR, waist‐to‐hip ratio.

## 1. Introduction

Steatotic liver disease (SLD) is prevalent worldwide, with rates of 25%, 44%, and 30% in Western Europe, Latin America, and Asia, respectively [1]. The SLD prevalence increased from 25.3% between 1990 and 2006 to 38% between 2016 and 2019 [2]. Hepatic steatosis may not cause immediate symptoms; however, excess fat accumulation can lead to inflammation. Persistent inflammation can result in severe liver diseases such as fibrosis, cirrhosis, and hepatocellular carcinoma (HCC). Studies have projected that between 2015 and 2030, the prevalence of cirrhosis and HCC associated with SLD is expected to increase by 168% and 137%, respectively [3]. Crucially, the progression of hepatic fibrosis in SLD is bidirectional [4], particularly in its earlier stages; this reversible ‘window of opportunity’ emphasizes the clinical necessity for early detection and lifestyle intervention [5].

Diagnosing SLD typically requires imaging techniques such as abdominal ultrasound, magnetic resonance imaging, computed tomography, or liver biopsy, which are not only costly and time‐intensive but also impractical for large‐scale applications. To address this, indices based on clinical data, such as anthropometric measurements and biochemical test results, have been developed to provide a more accessible approach to identifying SLD and evaluating it in large health datasets. Notable clinical indices, such as the NAFLD liver fat score (NAFLD‐LFS) [6], Framingham steatosis index (FSI) [7], hepatic steatosis index (HSI) [8], ZJU index [9], and fatty liver index (FLI) [10], were primarily calibrated based on the historical NAFLD framework. However, transitioning from NAFLD to the broader SLD nomenclature, introduced in 2023 as an umbrella term to encompass diverse etiologies, reveals a significant diagnostic gap. These legacy indices often exhibit suboptimal accuracy (AUROC 0.68–0.72) when applied to the refined SLD criteria, particularly failing to account for the metabolic heterogeneity inherent in the new definitions [11, 12]. This underscores the need for improved tools tailored to the evolving definition and scope of SLD, particularly those that account for sex, age, and other demographic variations in SLD prevalence and risk.

Our research focuses on creating and validating models to recognize SLD based on clinical data. Our indices are specifically engineered for research applications, facilitating the high‐throughput identification of SLD within large‐scale epidemiological datasets and enhancing the characterization of distinct disease phenotypes. They might also help screen for SLD in places with limited resources, acting as an alternative when tools like ultrasound are not available. However, these models are not meant for widespread screening, following guidelines from the American Association for the Study of Liver Diseases (AASLD). They are designed to supplement existing diagnostic methods and help target specific treatments.

Studies have revealed significant sex‐related differences in the prevalence of NAFLD. Innate effects attributable to sex and female sex hormones influence the risk profiles and disease phenotypes of NAFLD [13]. The prevalence of hepatic steatosis is significantly higher among the male population than among the female population [14]. However, its prevalence increases among postmenopausal women [15]. As circulating estradiol levels decline postmenopause, the subsequent metabolic shift significantly elevates the risk of hepatic steatosis [16]. The distinct metabolic trajectories driven by biological sex and menopausal status suggest that ‘one‐size‐fits‐all’ diagnostic tools may overlook critical, strata‐specific risk factors. Consequently, this study aimed to develop and validate three sex‐ and age‐stratified models tailored for men, women aged <55 years, and women aged ≥55 years, by integrating biomarkers such as *β*‐carotene and insulin that reflect these biological variances.

## 2. Methods

### 2.1. Study Sample

Participants were selected from the Third National Health and Nutrition Examination Survey (NHANES III), which collected cross‐sectional survey data from a sample of the United States population between 1988 and 1994 [17]. A multistage stratified sampling design was used to ensure that the results represented the health status of the noninstitutionalized civilian population of the United States. The operational and procedural details have been described previously [18]. A total of 33,994 individuals were surveyed. Participants were eligible for inclusion if they met all the following criteria: age 20 to 75 years, not pregnant, underwent an abdominal ultrasound examination with a gradable result, and fasted for more than 8 h before blood testing. Individuals with more than 20% missing baseline covariate data were excluded to reduce the influence of substantially incomplete baseline profiles on candidate predictor screening and multivariable model development. The 20% threshold was selected as a pragmatic data‐completeness criterion to balance data completeness, model stability, cohort retention, and statistical power during model development.

### 2.2. SLD Assessment

Three well‐trained ultrasound readers determined the primary finding of hepatic steatosis in the NHANES III based on defined criteria (https://wwwn.cdc.gov/nchs/data/nhanes3/34a/HGUHS.htm). During this study, SLD was classified according to the hepatic steatosis grade. A mild‐to‐severe grade was considered SLD‐positive, and a normal grade was considered SLD‐negative.

### 2.3. Clinical Data

The NHANES III encompassed the demographic, dietary, examination, laboratory, and questionnaire datasets. We extracted candidate variables, including demographic characteristics, physical measurements, and laboratory data. We excluded variables with more than 20% missing data, except for well‐known liver disease‐related factors, including plasma fibrinogen, serum lipoprotein(a), serum low‐density lipoprotein (LDL) cholesterol, and serum vitamin B12. We generated the following two new variables from the existing data: the homeostasis model assessment of insulin resistance (HOMA‐IR) index and aspartate aminotransferase (AST)/alanine aminotransferase (ALT) ratio. Data on 119 variables were included in the analysis. These variables were classified as demography, antibodies, biochemistry (serum), biochemistry (urine), cardiometabolic risk factors (CMRFs), criteria, diabetes mellitus (DM) tests, hematology, and medication (Table S1). For incomplete data, multivariate imputation was performed using chained equations in the R package “*mice*” [19].

### 2.4. External Validation

The NHANES 2017–2018 and Taiwan MJ Cohort (1996–2022) were used as external validation datasets for SSLDLD1, representing populations from the United States and Taiwan, respectively [17, 20]. The NHANES (2017–2018) comprises cross‐sectional health information, including demographics, physical examination results, laboratory data, and dietary information from individuals in the United States who were sampled between 2017 and 2018. Individuals were classified as the SLD group if their controlled attenuation parameter (CAP) according to liver ultrasound transient elastography was ≥ 238 dB/m [21]. Participants with available CAP data who met the four aforementioned inclusion criteria were included. Finally, 2545 participants from the NHANES (2017–2018) were enrolled for external validation.

Since 1996, the MJ Health Resource Center has collected health data from individuals who participated in a standard medical screening program at one of the three MJ Taiwan Health Evaluation Centers. The MJ Health datasets include demographics, lifestyle, medical history (recorded via questionnaire), laboratory and imaging data, and physical examination results from these medical screenings. SLD was defined based on the results of abdominal ultrasonography and the following diagnoses: fatty liver; mild fatty liver; moderate fatty liver; severe fatty liver; and alcoholic liver disease. A total of 20,592 individuals with imaging data who met the four aforementioned criteria were included. Participants in the NHANES (2017–2018) and MJ Cohort (1996–2022) provided informed consent, and both datasets received ethical approval.

### 2.5. Statistical Analysis

We developed sex‐ and age‐stratified SLD lab data (SLDLD) indices in NHANES III. External validation was subsequently performed for SSLDLD1 in NHANES 2017–2018 and the Taiwan MJ Health dataset. Women aged ≥ 55 years were treated as an age‐based proxy for postmenopausal status [22], consistent with prior epidemiologic studies. Continuous and categorical variables were summarized as means (standard deviations) and counts (percentages), and SLD prevalence was calculated.

A sex‐ and age‐stratified modeling pipeline was implemented (men, women aged < 55 years, and women aged ≥ 55 years). Within each stratum, variables associated with SLD were screened via an exposure‐wide association study (EWAS) using logistic regression adjusted for age (sex was not included because the EWAS was conducted within sex‐specific strata), applying Bonferroni correction for multiple comparisons [23]. Candidate predictors were shortlisted based on EWAS significance and dataset availability. To manage redundancy, when multiple candidate predictors were highly correlated (absolute pairwise correlation coefficient > 0.90), we retained one representative variable from each correlated cluster, prioritizing clinical interpretability. In addition, we avoided including mathematically coupled formulations of the same biological signal (e.g., AST, ALT, and AST/ALT ratio) simultaneously.

Final multivariable models were specified using stepwise selection guided by AIC, with clinical interpretability considered. In women aged < 55 years, we specified the simplified SSLDLD1 model without the AST/ALT ratio and retained ALT as the primary transaminase predictor to improve coefficient stability and interpretability and to avoid redundancy among mathematically dependent transaminase‐related predictors. This specification was supported by model‐fit comparison (AIC) and is documented in Table S2. To enhance clinical usability, SSLDLD models used categorical predictors defined by prespecified cut‐points.

Bootstrap internal validation was performed for SLDLD1 separately within each NHANES III derivation stratum (men, women aged < 55 years, and women aged ≥ 55 years) to examine internal validity and possible overfitting. For each of 1000 bootstrap resamples, the full SLDLD1 model‐building procedure, including stepwise AIC‐based variable selection from the original candidate set, was repeated. Apparent and test AUROCs were calculated for each resample, and optimism‐corrected AUROCs were obtained by subtracting the mean bootstrap optimism from the apparent AUROC of the original model. Predictor selection frequencies across bootstrap resamples were also summarized to assess model stability.

The added value of stratification was further examined by comparing the stratified SLDLD1 framework with a nonstratified pooled model derived from the full NHANES III derivation cohort. The pooled model was developed using predictors shared across the three derivation strata to avoid structural missingness and ensure fair comparison, and the final pooled model retained sex, *β*‐carotene, waist‐to‐hip ratio, HOMA‐IR, and AST/ALT ratio. For the stratified approach, each participant received a predicted probability from the corresponding stratum‐specific SLDLD1 model. For the pooled approach, all participants received a predicted probability from the same nonstratified model. Discrimination was compared in the overall cohort and within each subgroup using AUROC and paired DeLong tests based on predictions from the same individuals.

Clinical utility was further examined using decision curve analysis (DCA), in which SLDLD1 was compared with FLI, HSI, NAFLD‐LFS, and US‐FLI within each NHANES III derivation stratum. Net benefit was evaluated across threshold probabilities from 0.05 to 0.50. For indices that do not directly yield predicted probabilities, score values were mapped to predicted probabilities within the corresponding derivation stratum before DCA. Higher net benefit across a clinically relevant threshold range was interpreted as greater potential decision‐analytic utility.

Model discrimination was evaluated using AUROC and compared with FLI, HSI, NAFLD‐LFS, and US‐FLI [6, 8, 10, 12]. Paired DeLong tests were used for within‐sample AUROC comparisons based on predictions from the same individuals. Similar paired comparisons were used for feature‐reduction analyses (Table S7) and for implementation‐feasibility assessment of full SSLDLD1 versus an MJ‐available version excluding race, *β*‐carotene, and MCHC (Table S8). External validation of SSLDLD1 assessed AUROC and operating characteristics (sensitivity, specificity, PPV, and NPV) at prespecified cut‐points. All analyses were performed using R version 4.3.3 [24].

## 3. Results

**FIGURE 1:**
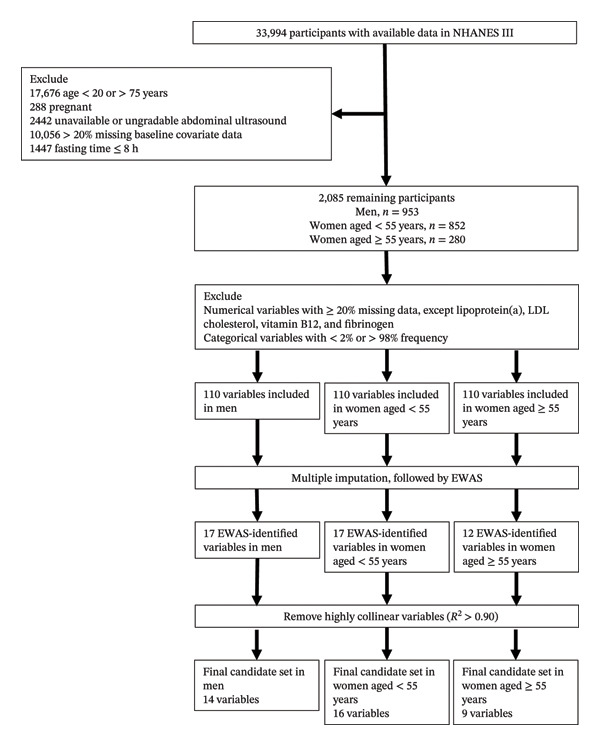
Study flow of the NHANES III derivation cohort and model‐development workflow. Participants were selected according to age, pregnancy status, availability of gradable abdominal ultrasound data, participant‐level baseline covariate completeness, and fasting time before blood testing. The final derivation cohort included 2085 participants stratified into men, women aged < 55 years, and women aged ≥ 55 years. After variable‐level filtering, multiple imputation, EWAS, and collinearity assessment, the final candidate sets included 14 variables in men, 16 variables in women aged < 55 years, and 9 variables in women aged ≥ 55 years.

The 2085 participants enrolled from NHANES III were separated into the following groups: men (*n* = 953), women aged < 55 years (*n* = 852), and women aged ≥ 55 years (*n* = 280) (Figure 1). Across these groups, most participants were White (range, 51%–67%). Compared with men, women had a slightly higher body mass index (BMI). Women aged ≥ 55 years had higher WC and higher total cholesterol, LDL, HDL, and TG levels than the other two strata. Men had the highest AST and ALT levels. The prevalence of SLD was 39% in men, 33% in women aged < 55 years, and 45% in women aged ≥ 55 years (Table 1).

**TABLE 1:** Baseline characteristics of the derivation (NHANES III) and external validation cohorts (NHANES 2017–2018 and MJ Health), stratified by sex and age.

Variable	NHANES III			NHANES 2017–2018			MJ health data		
	Men	Women < 55 years	Women ≥ 55 years	Men	Women < 55 years	Women ≥ 55 years	Men	Women < 55 years	Women ≥ 55 years
*N*	953	852	280	1245	722	578	9677	8962	1953
Age (y)	44.69 (15.88)	34.96 (9.55)	64.27 (5.52)	50.77 (17.51)	37.16 (10.13)	66.29 (7.92)	41.61 (12.57)	36.68 (8.44)	62.25 (5.85)
Race, %									
White	59	51	67	35	29	34	N/A	N/A	N/A
Black	36	45	30	23	23	25	N/A	N/A	N/A
Other	5	4	3	41	48	41	N/A	N/A	N/A
BMI (kg/m^2^)	26.75 (5.36)	28.23 (7.02)	28.84 (6.36)	29.28 (6.52)	30.58 (8.77)	30.17 (7.37)	24.81 (3.85)	21.91 (3.79)	24.01 (3.76)
WC (cm)	94.93 (14.35)	90.73 (16.35)	97.38 (14.35)	102.36 (16.43)	97.77 (19.34)	100.90 (15.54)	84.48 (9.73)	70.95 (8.38)	77.29 (8.53)
FPG (mg/dL)	103.91 (34.72)	96.43 (30.32)	111.15 (36.21)	108.24 (36.97)	96.56 (26.10)	110.88 (40.17)	105.52 (23.97)	97.06 (14.62)	109.96 (26.30)
HbA1c (%)	5.62 (1.19)	5.38 (1)	6.04 (1.3)	5.9 (1.13)	5.57 (0.83)	6.19 (1.29)	5.52 (0.87)	5.21 (0.59)	5.78 (0.91)
Total cholesterol (mg/dL)	202.49 (46.75)	191.21 (38.61)	236.49 (47.36)	184.51 (41)	183.5 (35.67)	200.29 (45.36)	197.98 (36.68)	190.26 (33.77)	212.01 (37.6)
LDL cholesterol (mg/dL)	126.93 (42.2)	116.82 (33.33)	143.98 (40.3)	157.05 (32.7)	151.18 (29.18)	171.21 (42.77)	121.29 (33.57)	106.34 (30.06)	122.87 (33.07)
HDL cholesterol (mg/dL)	47.83 (16.39)	52.21 (14.04)	56.58 (18.22)	48.75 (13.8)	56.5 (16.13)	59.19 (15.72)	52.52 (11.9)	65.15 (14.6)	63.18 (15.55)
Triglycerides (mg/dL)	152.39 (173.17)	117.9 (110.88)	165.62 (96.42)	144.82 (138.6)	110.26 (75.82)	130.74 (64.33)	134.08 (121.30)	81.93 (53.57)	117.18 (70.95)
AST (U/L)	26.63 (23.98)	20.1 (14.37)	21.19 (10.15)	23.98 (14.25)	19.02 (12.30)	20.89 (10.09)	26.36 (16.74)	20.37 (10.32)	26.41 (19.41)
ALT (U/L)	23.39 (23.13)	16.42 (14.15)	14.95 (8.74)	26.77 (19.01)	17.64 (12.83)	18.97 (11.18)	35.55 (28.47)	19.87 (17.91)	26.86 (24.89)
GGT (U/L)	47.71 (60.65)	31.56 (61.66)	34.99 (40.06)	37.72 (43.20)	23.99 (29.88)	30.66 (46.78)	39.67 (54.82)	19.9 (24.61)	28.3 (44.2)
ALP (U/L)	91.66 (34.90)	82.48 (29.52)	100.61 (33.26)	78.05 (25.27)	72.82 (24.18)	87.22 (27.05)	61.97 (19.78)	51.91 (17.4)	70.34 (26)
AST/ALT ratio	1.36 (0.72)	1.46 (0.64)	1.68 (1.56)	1.03 (0.40)	1.21 (0.39)	1.21 (0.37)	0.87 (0.33)	1.19 (0.36)	1.1 (0.31)
Hepatic steatosis (%)	39	33	45	70	56	70	53	24	52

### 3.1. EWAS Identification of Candidate Features

**FIGURE 2:**
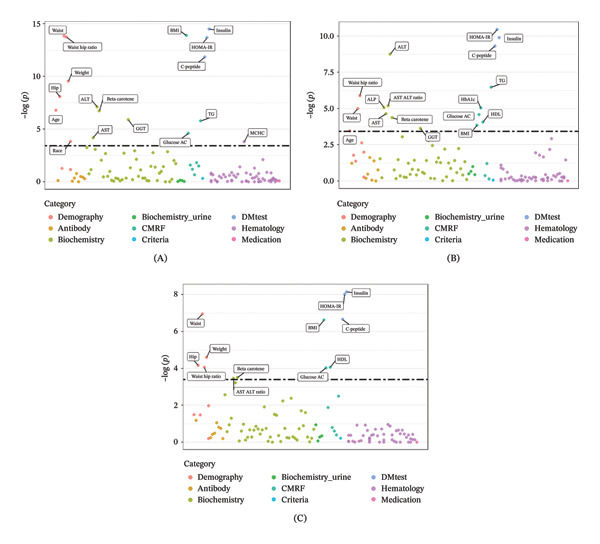
Exposure‐wide association study (EWAS) results across sex‐ and age‐stratified analyses. Panels (A)–(C) show EWAS results within each stratum: men, women aged < 55 years, and women aged ≥ 55 years, respectively. Models were adjusted for age, except when age itself was evaluated, and were Bonferroni corrected for multiple testing; features passing the significance threshold are labeled.

After age adjustment, except when age itself was evaluated, EWAS identified 17, 17, and 12 features significantly associated with SLD in men, women aged < 55 years, and women aged ≥ 55 years, respectively (Figure 2). Most identified features related to central adiposity and IR, including BMI, WC, WHR, insulin, HOMA‐IR, FPG, and C‐peptide, were positively associated with SLD. In men and women aged < 55 years, TG, gamma‐glutamyl transferase (GGT), ALT, AST, and age were associated with increased SLD risk. Weight and hip circumference were positively associated with SLD in men and women aged ≥ 55 years. Increased mean corpuscular hemoglobin concentration (MCHC) was associated with higher risk only in men, whereas glycated hemoglobin (HbA1c) and alkaline phosphatase (ALP) were identified only in women aged < 55 years. *β*‐Carotene was inversely associated with SLD in all three strata. In men, race was associated with SLD, with lower odds observed in the White reference category relative to other groups. The AST/ALT ratio and HDL were inversely associated in both female strata. No significant associations were observed in the biochemistry (urine), criteria, antibody, or medication domains (Figure 2).

### 3.2. Derivation of Stratified SLDLD Models

**FIGURE 3:**
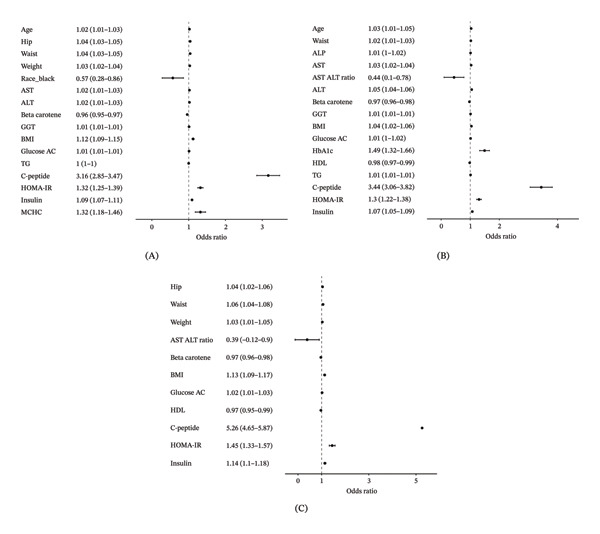
Multivariable associations of predictors included in the final SLDLD1 models across sex‐ and age‐stratified analyses. Panels (A)–(C) show effect estimates (odds ratios) with corresponding confidence intervals for men, women aged < 55 years, and women aged ≥ 55 years, respectively.

**FIGURE 4:**
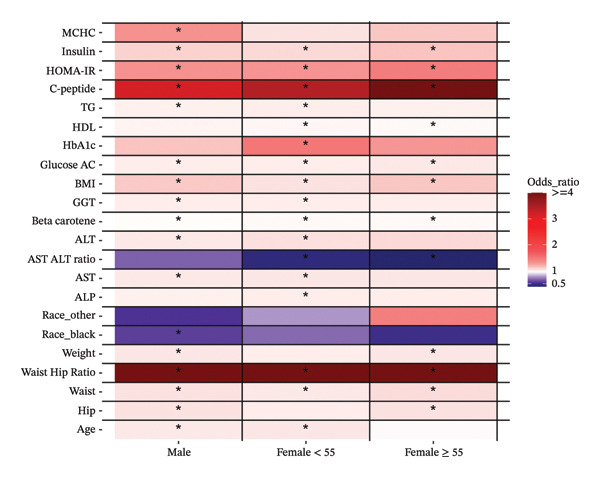
Heatmap summarizing the direction and relative magnitude of multivariable associations across strata. Red indicates positive associations and blue indicates inverse associations. Asterisks denote predictors retained in the final stratified SLDLD1 models. White race is the reference category where applicable.

After removing highly correlated variables (absolute pairwise correlation coefficient > 0.9), 14 candidate features remained for men, 16 for women aged < 55 years, and 9 for women aged ≥ 55 years (Table S2). Using these sex‐ and age‐stratified candidate sets, we developed two SLD indices, SLDLD1 and SLDLD2, via stepwise regression with a coefficient‐thresholding strategy (Table S2). SLDLD1 retained 9 predictors in men, 6 predictors in women aged < 55 years, and 4 predictors in women aged ≥ 55 years, whereas SLDLD2 was more compact, retaining 3 predictors in men and 4 predictors in each female stratum. In SLDLD1, insulin and *β*‐carotene were selected in all three strata. Age was included in men and women aged < 55 years, and BMI was included in men and women aged ≥ 55 years. Additional predictors in men included race, AST, GGT, FPG, and MCHC; in women aged < 55 years, ALT, HbA1c, and triglycerides were included; and in women aged ≥ 55 years, HDL was included (Table S2). The direction and magnitude of multivariable associations for predictors retained in the final SLDLD1 models are shown in Figure 3. A heatmap summarizing the direction and relative magnitude of these associations across strata is shown in Figure 4. For women aged < 55 years, the simplified SSLDLD1 model retained ALT and did not include the AST/ALT ratio (Table S2). For SLDLD2, WHR and C‐peptide were consistently selected across strata; race was included for men, the AST/ALT ratio was included for both female strata, and FPG was additionally included for women aged < 55 years. Nomograms for the full SLDLD1 and SLDLD2 models are shown in Figures S1–S3.

### 3.3. Internal Validation of SLDLD1

Bootstrap internal validation showed limited optimism across the three stratified SLDLD1 models (Table S4). In men, the apparent AUROC was 0.761 and the optimism‐corrected AUROC was 0.748, corresponding to a mean optimism of 0.014. In women aged < 55 years, the apparent and optimism‐corrected AUROCs were 0.709 and 0.689, respectively, with a mean optimism of 0.020. In women aged ≥ 55 years, the apparent AUROC was 0.791 and the optimism‐corrected AUROC was 0.767, with a mean optimism of 0.024. Predictor selection frequencies across 1000 bootstrap resamples indicated that model stability differed by stratum (Table S5). In men, age, race/ethnicity, *β*‐carotene, GGT, and BMI were selected consistently. In women aged < 55 years, ALT and *β*‐carotene were the most consistently retained variables, whereas several other predictors showed only moderate or low selection frequencies. In women aged ≥ 55 years, *β*‐carotene and BMI remained the most stable predictors, with insulin, HDL, AST/ALT ratio, and waist‐to‐hip ratio showing moderate selection stability.

### 3.4. Comparison With a Pooled Nonstratified Model

To further evaluate the added value of stratification, we compared the stratum‐specific SLDLD1 framework with a pooled nonstratified model derived from the full NHANES III derivation cohort. The pooled model was constructed using predictors shared across strata and retained sex, *β*‐carotene, waist‐to‐hip ratio, HOMA‐IR, and AST/ALT ratio (Table S6). The stratified approach showed better discrimination in the overall cohort (AUROC 0.751 vs 0.720; *p* < 0.001), in men (0.761 vs 0.726; *p* < 0.001), and in women aged < 55 years (0.709 vs 0.678; *p* = 0.013). In women aged ≥ 55 years, the stratified model remained numerically superior (0.791 vs 0.784), although the difference was not statistically significant (*p* = 0.650). These findings support the added value of sex‐ and age‐stratified model derivation, particularly in men and in women aged < 55 years.

### 3.5. Decision‐Analytic Performance

DCA further evaluated the potential clinical utility of SLDLD1 relative to existing indices (Figure S4). In men, SLDLD1 showed the highest net benefit across most clinically relevant threshold probabilities and consistently outperformed FLI, HSI, NAFLD‐LFS, and US‐FLI. In women aged < 55 years, SLDLD1 also showed the highest or near‐highest net benefit across most of the examined threshold range, generally exceeding FLI and US‐FLI and remaining slightly above or comparable to NAFLD‐LFS and HSI. In women aged ≥ 55 years, SLDLD1 again showed the highest or near‐highest net benefit, although the margin over FLI, HSI, and NAFLD‐LFS was smaller than that observed in men. Overall, the discrimination advantage of SLDLD1 was accompanied by favorable decision‐analytic performance in all three strata.

### 3.6. Simplified Implementation‐Oriented Models

To improve implementation feasibility, we developed simplified versions of the stratified models, denoted SSLDLD1 and SSLDLD2. In these implementation‐oriented models, selected predictors were coded using prespecified categorical rules, whereas BMI and WHR were retained as continuous predictors. The full SSLDLD equations and predictor coding rules are provided in Tables S9 and S10. The simplified SSLDLD models showed acceptable discrimination across strata (Table 2), although performance was modestly lower than that of the corresponding full SLDLD models. These results position SSLDLD as a simplified implementation‐oriented extension of the stratified SLDLD framework rather than a replacement for the full models.

**TABLE 2:** Discrimination performance (AUROC, 95% CI) of SLDLD/SSLDLD models compared with established steatosis indices in NHANES III subgroups.

Index	Men	Women < 55 years	Women ≥ 55 years
SLDLD1	0.76 [0.73, 0.79]	0.71 [0.67, 0.75]	0.79 [0.74, 0.84]
SLDLD2	0.71 [0.68, 0.75]	0.68 [0.64, 0.72]	0.72 [0.66, 0.78]
SSLDLD1	0.74 [0.71, 0.78]	0.66 [0.62, 0.70]	0.74 [0.69, 0.80]
SSLDLD2	0.70 [0.67, 0.74]	0.66 [0.62, 0.70]	0.68 [0.61, 0.74]
FLI	0.73 [0.69, 0.76]	0.67 [0.63, 0.71]	0.78 [0.72, 0.83]
HSI	0.71 [0.67, 0.74]	0.63 [0.58, 0.67]	0.74 [0.68, 0.80]
NAFLD‐LFS	0.67 [0.64, 0.71]	0.61 [0.57, 0.65]	0.73 [0.67, 0.79]
US‐FLI	0.70 [0.66, 0.73]	0.69 [0.65, 0.73]	0.76 [0.70, 0.81]

### 3.7. External Validation of SSLDLD1

We externally validated SSLDLD1 using samples from NHANES 2017–2018 and the MJ Health Database. Compared with NHANES III, the NHANES 2017–2018 cohort had a lower proportion of White participants and a higher proportion of individuals of other ethnicities. The three strata also had higher BMI, WC, LDL, and ALT levels and an approximately 1.5‐fold higher prevalence of hepatic steatosis (Table 1). The MJ Health Database, which includes mainly Asian individuals (> 90%), showed lower BMI, WC, and total cholesterol, LDL, and TG levels; however, it exhibited higher ALT levels and a greater prevalence of SLD among men and women aged ≥ 55 years.

### 3.8. Implementation Feasibility Under Predictor‐Availability Constraints

To adapt SSLDLD1 to the predictor availability of the MJ Health Database, we developed an MJ‐variables‐only version by excluding race, *β*‐carotene, and MCHC. In the NHANES cohorts, this restriction resulted in only small AUROC changes (Table S8), indicating that discrimination was largely preserved despite reduced predictor availability. In the MJ cohort, AUROC also remained high. However, fixed NHANES III‐derived probability thresholds produced a sensitivity‐specificity trade‐off, suggesting that discrimination was largely preserved across datasets, whereas threshold performance was not fully transferable. These findings indicate that dataset‐specific recalibration may be required before implementation in external populations (Table 3).

**TABLE 3:** Performance of SSLDLD1 across datasets using stratum‐specific NHANES III‐derived Youden cut‐points: discrimination (AUROC) and fixed‐threshold sensitivity/specificity, including the MJ‐variables‐only model.

Stratum	Dataset	AUROC [95% CI]	Sensitivity	Specificity	Threshold
Men	NHANES III	0.74 [0.71, 0.78]	0.68	0.73	0.383
	NHANES 2017‐2018	0.80 [0.77, 0.82]	0.68	0.74	0.383
	NHANES III (MJ variables only)	0.73 [0.70, 0.77]	0.74	0.63	0.334
	MJ Health dataset	0.79 [0.78, 0.80]	0.57	0.82	0.334

Women < 55 years	NHANES III	0.66 [0.62, 0.70]	0.51	0.77	0.335
	NHANES 2017‐2018	0.78 [0.74, 0.81]	0.58	0.84	0.335
	NHANES III (MJ variables only)	0.66 [0.62, 0.70]	0.51	0.78	0.337
	MJ Health dataset	0.74 [0.73, 0.75]	0.38	0.93	0.337

Women ≥ 55 years	NHANES III	0.74 [0.69, 0.80]	0.66	0.76	0.44
	NHANES 2017‐2018	0.71 [0.67, 0.76]	0.49	0.82	0.44
	NHANES III (MJ variables only)	0.74 [0.68, 0.80]	0.66	0.76	0.44
	MJ Health dataset	0.80 [0.78, 0.82]	0.13	0.98	0.44

*Note:* Thresholds were derived in NHANES III and applied unchanged to external datasets; differences in sensitivity and specificity reflect calibration and case‐mix differences rather than discrimination alone. AUROC indicates the area under the receiver operating characteristic curve and is presented with 95% confidence intervals (CIs) estimated using DeLong’s method. Sensitivity and specificity were calculated at a fixed probability threshold. Thresholds were derived in the NHANES III derivation dataset within each stratum using the Youden index and then applied unchanged for evaluation in each dataset. The NHANES III and NHANES 2017–2018 rows correspond to the full SSLDLD1 model, whereas the “NHANES III (MJ variables only)” and MJ Health dataset rows correspond to the MJ‐variables‐only version of SSLDLD1. For the MJ‐variables‐only model, the stratum‐specific threshold was derived in NHANES III using MJ‐variables‐only predictions and then applied unchanged to the MJ Health dataset. Sample size and SLD prevalence are reported in Table 1.

### 3.9. Feature Reduction Analysis

Because insulin and *β*‐carotene are not routinely measured in many general screening settings, we further evaluated the effect of removing these nonroutine biomarkers from the models. As shown in Table S7, omission of insulin and/or β‐carotene resulted in only small AUROC changes across most strata. For example, removing insulin in women aged < 55 years did not result in a statistically significant AUROC change (*p* = 0.4763), and removing *β*‐carotene in women aged ≥ 55 years also did not result in a statistically significant AUROC change (*p* = 0.0987). In men, exclusion of β‐carotene (*p* = 0.0106) or simultaneous exclusion of insulin and *β*‐carotene (*p* = 0.0131) led to a small but statistically significant reduction in AUROC, although the absolute decrease was modest (0.0133). These findings support the feasibility of reduced‐predictor implementation when specific biomarkers are unavailable, while acknowledging a modest performance trade‐off in men when *β*‐carotene is omitted.

## 4. Discussion

In this study, we identified distinct SLD profiles across three demographic strata and developed sex‐ and age‐stratified, laboratory‐based indices for SLD identification, including the high‐precision SLDLD and the simplified categorical version, SSLDLD. In head‐to‐head comparisons using paired DeLong tests, SLDLD1 significantly outperformed established indices such as FLI and HSI across men, women aged < 55 years, and women aged ≥ 55 years (Table S3). When compared with NAFLD‐LFS and US‐FLI, SLDLD1 showed numerically comparable or higher AUROC values across strata, although the magnitude of improvement varied by subgroup and some differences were not statistically significant. Overall, these findings support a stratified, laboratory‐based framework for SLD identification, with SLDLD1 serving as the principal high‐discrimination model and SSLDLD as a more clinically accessible complementary version [6, 8, 10, 12]. Beyond conventional head‐to‐head AUROC comparison, the originality of this study lies in integrating sex‐ and age‐stratified model derivation, bootstrap‐based model stability assessment, direct comparison against a pooled nonstratified model, DCA, and cross‐dataset implementation testing under predictor‐availability constraints.

DCA further supported the potential clinical relevance of SLDLD1. Across the three strata, SLDLD1 generally showed the highest or near‐highest net benefit over clinically relevant threshold probabilities, indicating that its discriminatory advantage was accompanied by favorable decision‐analytic performance. This pattern was most pronounced in men, remained evident in women aged < 55 years, and was more modest in women aged ≥ 55 years, broadly consistent with the subgroup differences observed in the bootstrap and pooled‐versus‐stratified analyses.

The rationale for stratification is supported by heterogeneity in metabolic phenotypes and fat distribution across sex and age. Across all strata, obesity‐related measures, especially WHR, WC, and BMI, were consistently associated with SLD, reinforcing the central role of adiposity. Markers of IR and glycemic dysregulation, including FPG, C‐peptide, HOMA‐IR, and insulin, were also associated with SLD, emphasizing their close link with hepatic fat accumulation. In contrast, *β*‐carotene was inversely associated with SLD across all strata and may reflect broader metabolic, nutritional, or antioxidant‐related status. The final predictor composition of SLDLD1 also differed meaningfully across strata. In men, the model retained age, race/ethnicity, AST, *β*‐carotene, GGT, BMI, FPG, insulin, and MCHC. In women aged < 55 years, the final model included age, ALT, *β*‐carotene, HbA1c, triglycerides, and insulin. In women aged ≥ 55 years, the model was further simplified to *β*‐carotene, BMI, HDL, and insulin. These differences are biologically plausible and support sex‐ and age‐specific model derivation [13, 16].

The value of stratification was further supported by direct comparison with a nonstratified pooled model derived from the full NHANES III derivation cohort. The pooled model retained sex, *β*‐carotene, waist‐to‐hip ratio, HOMA‐IR, and AST/ALT ratio, thereby representing a common predictor set applicable across strata. Against this pooled model, the stratified SLDLD1 framework showed better overall discrimination and improved subgroup‐specific discrimination in men and women aged < 55 years, whereas in women aged ≥ 55 years the stratified model remained numerically superior, but the gain was small. These findings indicate that stratification adds empirical value beyond biological plausibility, particularly in subgroups with more distinct hepatic and metabolic predictor patterns.

Bootstrap internal validation further informed the internal robustness and subgroup‐specific stability of the stratified SLDLD1 models. Across 1000 bootstrap resamples, the optimism‐corrected AUROCs remained close to the apparent AUROCs in men and women aged ≥ 55 years, indicating only modest optimism. In women aged < 55 years, the corrected AUROC was lower, suggesting comparatively more modest discrimination in this subgroup. Predictor selection frequencies showed a similar pattern. In men, age, race/ethnicity, *β*‐carotene, GGT, and BMI were selected consistently, indicating a relatively stable core predictor set. In women aged ≥ 55 years, *β*‐carotene and BMI also showed high selection stability, with insulin, HDL, AST/ALT ratio, and WHR demonstrating moderate robustness. By contrast, in women aged < 55 years, only ALT and *β*‐carotene were retained with very high frequency, whereas several other predictors were more sample‐sensitive. Taken together, these findings suggest that the younger female subgroup may have a more heterogeneous biomarker‐based pattern and less stable predictor structure and should therefore be interpreted with somewhat greater caution.

A practical strength of this work is that the stratified indices can be positioned for settings with different levels of data availability. SLDLD1 offers higher discrimination and may be most suitable for research phenotyping or data‐rich clinical environments, whereas SSLDLD1 and the reduced‐predictor versions may have greater real‐world applicability in routine screening or health‐check databases where *β*‐carotene and insulin are not commonly available. Because biomarkers such as *β*‐carotene and insulin are not routinely measured in many general screening or primary care settings, we additionally examined reduced‐predictor performance. Feature‐reduction analyses showed that omitting insulin and/or *β*‐carotene resulted in only small AUROC changes across most strata. In men, exclusion of *β*‐carotene, or simultaneous exclusion of insulin and *β*‐carotene, produced a statistically significant but small absolute reduction in AUROC, indicating that the performance impact was modest but not identical across subgroups. These findings suggest that the full models may remain preferable when complete biomarker data are available, whereas simplified or reduced‐predictor implementations may be more feasible for broad deployment, with an acceptable but context‐dependent trade‐off between discrimination and implementation feasibility.

External validation of SSLDLD1 in NHANES 2017–2018 and the MJ Health Database supported external discrimination across populations with different ethnic compositions, metabolic profiles, and SLD ascertainment methods. To facilitate application in the MJ dataset, we constructed an MJ‐variables‐only version of SSLDLD1 by excluding race, *β*‐carotene, and MCHC. Restricting the model to MJ‐available variables produced small absolute AUROC changes overall in the NHANES cohorts, although a statistically significant reduction was observed in men in NHANES 2017–2018. In the MJ cohort, discrimination also remained acceptable to good. However, fixed NHANES III‐derived probability thresholds did not transfer optimally to MJ and resulted in a sensitivity‐specificity trade‐off, particularly in women aged ≥5. These findings suggest that ranking performance was partly preserved, whereas threshold‐level performance was influenced by differences in case mix, predictor availability, and outcome definitions across datasets. Therefore, the external validation results should be interpreted as evidence of cross‐cohort discriminatory performance rather than proof of direct clinical transportability, and direct transfer of fixed probability thresholds across populations or clinical settings should be approached with caution.

## 5. Limitations

Several limitations should be considered. First, ultrasound in the development cohort has limited sensitivity for mild steatosis, which may have introduced outcome misclassification. Second, women aged ≥ 55 years were treated as an age‐based proxy for postmenopausal status, which may have introduced misclassification. Third, because C‐peptide was unavailable in both external validation datasets, we were unable to externally validate the more compact SLDLD2/SSLDLD2 models. External validation was therefore limited to SSLDLD1, which was selected a priori as the implementation‐oriented model for cross‐dataset testing. Fourth, although bootstrap internal validation supported overall model robustness, the lower optimism‐corrected AUROC and less stable predictor structure in women aged < 55 years suggest that this subgroup may require further refinement in future studies. Fifth, the 20% missingness threshold was used as a pragmatic preprocessing criterion, recognizing that no universally accepted participant‐level cutoff exists for baseline covariate missingness. Because this threshold was part of the predefined preprocessing workflow for model derivation, we did not perform a separate post hoc re‐derivation using a stricter participant‐level missing‐data threshold, such as 10%. Therefore, the sensitivity of the full model‐development workflow to alternative participant‐level missing‐data thresholds was not directly quantified. Finally, external validation cohorts differed not only in population characteristics but also in SLD ascertainment, including ultrasound‐based steatosis in NHANES III, CAP‐based steatosis in NHANES 2017–2018, and ultrasound‐derived diagnostic categories in the MJ Health Database. Such cross‐cohort heterogeneity may affect comparability, calibration, and threshold performance, and therefore limit overgeneralization of fixed cut‐points across populations and clinical settings. Future work should prioritize population‐specific recalibration and further validation of decision thresholds across diverse clinical settings.

## 6. Conclusion

In conclusion, we developed a sex‐ and age‐stratified, laboratory‐based framework for SLD identification. SLDLD1 significantly outperformed established markers such as FLI and HSI across all three demographic strata in derivation, whereas SSLDLD1 preserved good discrimination across U.S. and Asian external datasets and remained competitive under restricted predictor availability. This flexible toolbox, comprising a high‐precision research‐oriented model and more user‐friendly categorical versions, may support large‐scale epidemiological studies and practical SLD identification in settings where advanced imaging is unavailable [6, 8, 10].

Bootstrap‐based internal validation showed only modest optimism in the stratified SLDLD1 models, supporting their overall internal robustness, although the model for women aged < 55 years showed comparatively more modest corrected discrimination and should be interpreted with greater caution. Feature‐reduction analyses also showed minimal AUROC change after excluding nonroutine biomarkers in most strata, with only a modest performance decrease in men when *β*‐carotene was removed. These findings support SSLDLD1 and reduced‐predictor versions as practical implementation‐oriented tools when nonroutine biomarkers are unavailable. However, because external cohorts differed in population structure, predictor availability, and SLD ascertainment, external implementation should emphasize local recalibration and validation of decision thresholds rather than direct transfer of fixed cut‐points across settings [1, 11].

## Author Contributions

Weiting Liaw: methodology, software, formal analysis, investigation, data curation, visualization, and writing–original draft.

Pei‐Chien Tsai: data curation, validation, and writing–review and editing.

Ming‐Lung Yu: conceptualization, methodology, resources, supervision, and writing–review and editing.

I‐Jung Feng: conceptualization, methodology, software, data curation, formal analysis, validation, supervision, project administration, funding acquisition, and writing–review and editing.

## Funding

This study was supported by the Ministry of Education (MOE), Taiwan, under the Higher Education Sprout Project (Center of Excellence for Metabolic Associated Fatty Liver Disease, National Sun Yat‐sen University) and by the NSYSU‐KMU Joint Research Project (Grant No. NSYSUKMU114‐P28).

## Disclosure

All authors critically reviewed the manuscript, approved the final version, and agreed to be accountable for all aspects of the work.

## Ethics Statement

This study was approved by the Institutional Review Board of Chi Mei Medical Center (Approval No. 11301‐E04). NHANES III and NHANES 2017–2018 are publicly available, deidentified datasets, and written informed consent was obtained from participants during the original NHANES data collection. For the Taiwan MJ Health Database, participants provided informed consent for the use of deidentified health‐screening data for research purposes. All datasets used in this study were deidentified before release to the investigators and before analysis. The study was conducted in accordance with the Declaration of Helsinki and relevant local ethical guidelines.

## Conflicts of Interest

The authors declare no conflicts of interest.

## Supporting Information

Additional supporting information can be found online in the Supporting Information section.

## Supporting information


**Supporting Information** TABLE S1. Variables screened across the nine feature domains in the EWAS. TABLE S2. Predictor composition for SLDLD and SSLDLD indices across sex and age strata. TABLE S3. Paired DeLong tests comparing AUROC of SLDLD1 versus established steatosis indices. TABLE S4. Bootstrap internal validation of the stratified SLDLD1 models in the NHANES III derivation cohort. TABLE S5. Predictor selection frequencies of SLDLD1 across 1000 bootstrap resamples in the NHANES III derivation cohort. TABLE S6. Comparison of a pooled nonstratified SLDLD1 model and the stratified SLDLD1 framework in the NHANES III derivation cohort. TABLE S7. Impact of feature reduction on AUROC of SLDLD1 and SSLDLD1 in the NHANES III derivation cohort. TABLE S8. Portability assessment of SSLDLD1 after restricting predictors to variables available in the MJ Health Database. TABLE S9. Full equations and predictor composition for SSLDLD models across sex and age strata. TABLE S10. Coding definitions for predictors used in the SSLDLD models. FIGURE S1. Nomograms for men. (A) SLDLD1. (B) SLDLD2. FIGURE S2. Nomograms for women aged < 55 years. (A) SLDLD1. (B) SLDLD2. FIGURE S3. Nomograms for women aged ≥ 55 years. (A) SLDLD1. (B) SLDLD2. FIGURE S4. Decision curve analysis comparing SLDLD1 with existing indices across the three NHANES III derivation strata.

## Data Availability

The NHANES III and NHANES 2017‐2018 datasets used in this study are publicly available through the National Center for Health Statistics. The Taiwan MJ Health Database is a third‐party dataset and is not publicly available because of data‐use restrictions and participant privacy protections. Part of the data used in this research was authorized by, and received from, the MJ Health Research Foundation (Authorization Code: MJHRF2024012A). Any interpretation or conclusion described in this paper does not represent the views of the MJ Health Research Foundation. Access to MJ Health data may be requested from the MJ Health Research Foundation, subject to institutional approval and data‐use agreements.
