# Supplementary material for: Development and Validation of the Steatotic Liver Disease Lab Data Index: A Refined Diagnostic Tool
Source: Can J Gastroenterol Hepatol. 2026 Jun 30;2026:7366206. doi: 10.1155/cjgh/7366206 (PMC13316952; doi:10.1155/cjgh/7366206)
Supplement: Supplementary file 1 — Supporting Information TABLE S1. Variables screened across the nine feature domains in the EWAS. TABLE S2. Predictor composition for SLDLD and SSLDLD indices across sex and age strata. TABLE S3. Paired DeLong tests comparing AUROC of SLDLD1 versus established steatosis indices. TABLE S4. Bootstrap internal validation of the stratified SLDLD1 models in the NHANES III derivation cohort. TABLE S5. Predictor selection frequencies of SLDLD1 across 1000 bootstrap resamples in the NHANES III derivation cohort. TABLE S6. Comparison of a pooled nonstratified SLDLD1 model and the stratified SLDLD1 framework in the NHANES III derivation cohort. TABLE S7. Impact of feature reduction on AUROC of SLDLD1 and SSLDLD1 in the NHANES III derivation cohort. TABLE S8. Portability assessment of SSLDLD1 after restricting predictors to variables available in the MJ Health Database. TABLE S9. Full equations and predictor composition for SSLDLD models across sex and age strata. TABLE S10. Coding definitions for predictors used in the SSLDLD models. FIGURE S1. Nomograms for men. (A) SLDLD1. (B) SLDLD2. FIGURE S2. Nomograms for women aged < 55 years. (A) SLDLD1. (B) SLDLD2. FIGURE S3. Nomograms for women aged ≥ 55 years. (A) SLDLD1. (B) SLDLD2. FIGURE S4. Decision curve analysis comparing SLDLD1 with existing indices across the three NHANES III derivation strata. [file CJGH-2026-7366206-s001.zip › 7366206_Supporting_Information_Tables_S1-S10 (2) (1).docx]

**TABLE S1. Variables screened across the nine feature domains in the EWAS.**

| **Categories** | **Features** |
| --- | --- |
| Demography | Sex, age, race, weight, height, smoking, alcohol, waist, and waist-to-hip ratio (n=9) |
| Antibody | Hepatitis A antibody, hepatitis B core antibody, hepatitis B surface antibody, serum hepatitis B surface antigen, hepatitis C antibody, hepatitis D antibody, herpes I antibody, serum herpes II antibody, rubella antibody, varicella antibody, toxoplasmosis antibody, rheumatoid factor antibody, latex antibody, *Helicobacter pylori* antibody (n=14) |
| Biochemistry (serum) | Free erythrocyte porphyrins, iron, total iron-binding capacity, transferrin saturation, ferritin, folate, RBC folate, vitamin B12, vitamin C, calcium, selenium, vitamin A, vitamin E, α-carotene, *β*-carotene, *β*-cryptoxanthin, lutein/zeaxanthin, lycopene, sum retinyl esters, cotinine, cholesterol, triglycerides, LDL, apolipoprotein AI, apolipoprotein B, lipoprotein, follicle-stimulating hormone, luteinizing hormone, fibrinogen, C-reactive protein, sodium, potassium, chloride, HCO_3_, phosphorus, urinary albumin, blood urea nitrogen, bilirubin, creatinine, AST, ALT, γ-glutamyl transferase, lactate dehydrogenase, ALP, total protein, albumin, globulin, osmolality, AST/ALT ratio (n=48) |
| Biochemistry (urine) | Cadmium, creatinine, albumin, iodine (n=4) |
| CMRF | BMI, HbA1c, FPG, triglycerides, HDL, blood pressure (n=6) |
| Criteria | Pregnancy, fasting hour (n=2) |
| DM tests | C-peptide, insulin, HOMA-IR* (n=3) |
| Hematology | White blood cells, lymphocyte, mononuclear, granulocyte, red blood cells, hemoglobin, hematocrit, mean corpuscular volume, MCHC, red blood cell distribution width, platelet, platelet distribution width, mean platelet volume, segmented neutrophils, lymphocytes, monocytes, eosinophils, basophils, blasts, promyelocytes, metamyelocytes, myelocytes, bands, atypical lymphocytes, anisocytosis, basophilic stippling, hypochromia, poikilocytosis, polychromatophilia, macrocytosis, sickle cells, spherocytosis, target cells, toxic granulation, vacuolated cells (n=35) |
| Medication | Insulin (n=1) |

Candidate variables screened in the EWAS, grouped into nine predefined domains. HOMA-IR was calculated as fasting plasma glucose (mg/dL) × insulin (mIU/L) / 405.

**TABLE S2. Predictor composition for SLDLD and SSLDLD indices across sex and age strata.**

| **Category** | **Variable** | **SLDLD1/ SSLDLD1** | | | **SLDLD2/ SSLDLD2** | | |
| --- | --- | --- | --- | --- | --- | --- | --- |
|  |  | Men | Women <55 years | Women ≥55 years | Men | Women <55 years | Women ≥55 years |
| Demography | Age | **🗸** | **🗸** |  |  |  |  |
| Demography | Race | **🗸** |  |  | **🗸** |  |  |
| Demography | WHR |  |  |  | **🗸** | **🗸** | **🗸** |
| Biochemistry | AST | **🗸** |  |  |  |  |  |
| Biochemistry | ALT |  | **🗸** |  |  |  |  |
| Biochemistry | AST/ALT ratio |  | * |  |  | **🗸** | **🗸** |
| Biochemistry | *β*-carotene | **🗸** | **🗸** | **🗸** |  |  |  |
| Biochemistry | GGT | **🗸** |  |  |  |  |  |
| CMRF | BMI | **🗸** |  | **🗸** |  |  |  |
| CMRF | FPG | **🗸** |  |  |  | **🗸** |  |
| CMRF | HbA1c |  | **🗸** |  |  |  |  |
| CMRF | TG |  | **🗸** |  |  |  |  |
| CMRF | HDL |  |  | **🗸** |  |  |  |
| DM test | Insulin | **🗸** | **🗸** | **🗸** |  |  |  |
| DM test | C-peptide |  |  |  | **🗸** | **🗸** | **🗸** |
| Hematology | MCHC | 🗸 |  |  |  |  |  |
| No. of features | | **9** | **6** | **4** | **3** | **4** | **4** |

Predictor composition of the stratified SLDLD and SSLDLD models in men, women aged <55 years, and women aged ≥55 years. Model 1 includes insulin, whereas Model 2 includes C-peptide. Final SSLDLD equations and coding rules are provided in Tables S9 and S10.

**TABLE S3. Paired DeLong tests comparing AUROC of SLDLD1 versus established steatosis indices.**

| **Stratum** | **Comparator index** | **AUROC of SLDLD1 [95% CI]** | **AUROC of Comparator [95% CI]** | **ΔAUROC (SLDLD1 vs. Comparator)** | **Paired DeLong’s P-value** |
| --- | --- | --- | --- | --- | --- |
| Men | FLI | 0.76 [0.73, 0.79] | 0.71 [0.67, 0.74] | 0.054 | **<0.001** |
|  | HSI |  | 0.67 [0.64, 0.71] | 0.089 | **<0.001** |
|  | NAFLD‑LFS |  | 0.70 [0.66, 0.73] | 0.063 | **<0.001** |
|  | US-FLI |  | 0.73 [0.69, 0.76] | 0.034 | **<0.001** |
| Women <55 years | FLI | 0.71 [0.67, 0.75] | 0.63 [0.58, 0.67] | 0.083 | **<0.001** |
|  | HSI |  | 0.61 [0.57, 0.65] | 0.099 | **<0.001** |
|  | NAFLD‑LFS |  | 0.69 [0.65, 0.73] | 0.016 | 0.1817 |
|  | US-FLI |  | 0.67 [0.63, 0.71] | 0.035 | **0.0053** |
| Women ≥55 years | FLI | 0.79 [0.74, 0.84] | 0.74 [0.68, 0.80] | 0.052 | **0.0125** |
|  | HSI |  | 0.73 [0.67, 0.79] | 0.061 | **0.0062** |
|  | NAFLD‑LFS |  | 0.76 [0.70, 0.81] | 0.034 | 0.0616 |
|  | US-FLI |  | 0.78 [0.72, 0.83] | 0.015 | 0.3840 |

Pairwise AUROC comparisons between SLDLD1 and established comparator indices in each derivation stratum. ΔAUROC was calculated as AUROC(SLDLD1) minus AUROC(comparator). *P*-values are two-sided.

**TABLE S4. Bootstrap internal validation of the stratified SLDLD1 models in the NHANES III derivation cohort.**

| **Stratum** | **Men** | **Women aged <55 years** | **Women aged ≥55 years** |
| --- | --- | --- | --- |
| n | 953 | 852 | 280 |
| Bootstrap resamples requested | 1000 | 1000 | 1000 |
| Valid bootstrap resamples | 1000 | 1000 | 1000 |
| Apparent AUROC | 0.7610 | 0.7087 | 0.7911 |
| Mean bootstrap apparent AUROC | 0.7669 | 0.7234 | 0.8087 |
| Mean bootstrap test AUROC | 0.7533 | 0.7033 | 0.7848 |
| Mean optimism | 0.0135 | 0.0200 | 0.0240 |
| Optimism-corrected AUROC | 0.7475 | 0.6886 | 0.7672 |
| Original selected SLDLD1 model | Age + Race/ethnicity + AST + β-carotene + GGT + BMI + FPG + Insulin + MCHC | Age + ALT + β-carotene + HbA1c + Triglycerides + Insulin | β-carotene + BMI + HDL + Insulin |

Bootstrap internal validation results for the stratum-specific SLDLD1 models across 1,000 resamples. Optimism-corrected AUROC was calculated as the apparent AUROC minus mean optimism.

**TABLE S5. Predictor selection frequencies of SLDLD1 across 1,000 bootstrap resamples in the NHANES III derivation cohort.**

**Panel A. Men**

| **Predictor** | **Selection frequency** |
| --- | --- |
| β-carotene | 0.991 |
| BMI | 0.951 |
| Age | 0.945 |
| GGT | 0.911 |
| Race/ethnicity | 0.820 |
| Insulin | 0.657 |
| AST | 0.626 |
| MCHC | 0.585 |
| FPG | 0.581 |
| HOMA-IR | 0.367 |
| ALT | 0.346 |
| Waist-to-hip ratio | 0.297 |
| Triglycerides | 0.176 |
| C-peptide | 0.127 |

**Panel B. Women aged <55 years**

| **Predictor** | **Selection frequency** |
| --- | --- |
| ALT | 0.982 |
| β-carotene | 0.963 |
| Triglycerides | 0.797 |
| Age | 0.749 |
| HbA1c | 0.550 |
| Insulin | 0.538 |
| HOMA-IR | 0.493 |
| GGT | 0.417 |
| HDL | 0.377 |
| BMI | 0.375 |
| FPG | 0.375 |
| AST | 0.273 |
| Waist-to-hip ratio | 0.223 |
| ALP | 0.212 |
| C-peptide | 0.184 |

**Panel C. Women aged ≥55 years**

| **Predictor** | **Selection frequency** |
| --- | --- |
| β-carotene | 0.942 |
| BMI | 0.819 |
| Insulin | 0.640 |
| HDL | 0.568 |
| AST/ALT ratio | 0.559 |
| Waist-to-hip ratio | 0.553 |
| C-peptide | 0.423 |
| FPG | 0.386 |
| HOMA-IR | 0.375 |

Selection frequency of predictors retained in the final SLDLD1 model across 1,000 bootstrap resamples, stratified by sex and age group.

**TABLE S6. Comparison of a pooled non-stratified SLDLD1 model and the stratified SLDLD1 framework in the NHANES III derivation cohort.**

| **Evaluation set** | **n** | **Pooled model AUROC** | **Stratified model AUROC** | **AUROC difference** | **Paired DeLong P** | **Preferred approach** |
| --- | --- | --- | --- | --- | --- | --- |
| **Overall cohort** | 2085 | 0.7202 | 0.7512 | 0.0310 | < 0.0001 | Stratified |
| **Men** | 953 | 0.7256 | 0.7610 | 0.0354 | 0.0002 | Stratified |
| **Women aged <55 years** | 852 | 0.6782 | 0.7087 | 0.0305 | 0.0134 | Stratified |
| **Women aged ≥55 years** | 280 | 0.7836 | 0.7911 | 0.0075 | 0.6504 | Stratified |

Comparison of discrimination between a pooled non-stratified SLDLD1 model and the stratified SLDLD1 framework in NHANES III. The pooled model was derived in the full NHANES III derivation cohort using predictors shared across strata to avoid structural missingness and ensure fair comparison. The final pooled model retained sex, *β*-carotene, waist-to-hip ratio, HOMA-IR, and AST/ALT ratio. AUROC difference was calculated as stratified minus pooled AUROC.

**TABLE S7. Impact of feature reduction on AUROC of SLDLD1 and SSLDLD1 in the NHANES III derivation dataset**

| **Stratum** | **Model** | **Full Model AUROC** | **Excl. Insulin AUROC (*P*)** | **Excl. *β*-carotene AUROC (*P*)** | **Excl. Both AUROC (*P*)** |
| --- | --- | --- | --- | --- | --- |
| Men | SLDLD1 | 0.7610 | 0.7597 (*P* = 0.2314) | 0.7477 (*P* = **0.0106**) | 0.7481 (*P*= **0.0131**) |
|  | SSLDLD1 | 0.7449 | 0.7380 (*P* = 0.1836) | 0.7450 (*P* = 0.6010) | 0.7410 (*P* = 0.4841) |
| Women <55 years | SLDLD1 | 0.7087 | 0.7098 (*P* = 0.4763) | 0.7015 (*P* = 0.3494) | 0.7039 (*P* = 0.5423) |
|  | SSLDLD1 | 0.6598 | 0.6595 (*P* = 0.9780) | 0.6632 (*P* = 0.7483) | 0.6617 (*P* = 0.8994) |
| Women ≥55 years | SLDLD1 | 0.7911 | 0.7950 (*P* = 0.7182) | 0.7720 (*P* = 0.0987) | 0.7757 (*P* = 0.2181) |
|  | SSLDLD1 | 0.7431 | 0.7437 (*P* = 0.9790) | 0.7550 (*P* = 0.3062) | 0.7461 (*P* = 0.8636) |

Effect of excluding insulin, β-carotene, or both from the full SLDLD1 and SSLDLD1 models in each derivation stratum. Reduced models were compared with the corresponding full model using paired DeLong tests.

**TABLE S8. Portability assessment of SSLDLD1 after restricting predictors to variables available in the MJ Health Database.**

| **Stratum** | **Dataset** | **AUROC (full)** | **AUROC (MJvars)** | **ΔAUROC (MJvars - full)** | **Paired DeLong *P*-value** |
| --- | --- | --- | --- | --- | --- |
| Men | NHANES 2017-2018 | 0.8 | 0.79 | -0.009 | **0.0372** |
|  | NHANES III | 0.74 | 0.73 | -0.010 | 0.0929 |
| Women <55 years | NHANES 2017-2018 | 0.78 | 0.78 | 0.001 | 0.4212 |
|  | NHANES III | 0.66 | 0.66 | -0.002 | 0.5719 |
| Women ≥55 years | NHANES 2017-2018 | 0.71 | 0.72 | 0.007 | 0.0631 |
|  | NHANES III | 0.74 | 0.74 | 0.000 | 0.2342 |

Comparison of the full SSLDLD1 model and an MJ-variables-only version in NHANES III and NHANES 2017–2018. ΔAUROC was calculated as AUROC(MJ variables only) minus AUROC(full).

**TABLE S9. Full equations and predictor composition for SSLDLD models across sex and age strata.**

SSLDLD equations for each stratum and model are provided. Predicted probability was calculated as $\frac{e^{LP}}{1+e^{LP}}$, where LP denotes the linear predictor. AUROC values correspond to the derivation performance of each SSLDLD model in NHANES III. Coding definitions for categorized predictors are provided in **Table S10**. The coefficient for β-carotene in women aged ≥55 years, SSLDLD1 should be interpreted cautiously because of instability in estimation.

| **Stratum / Model** | **Linear predictor (LP)** | **AUROC** |
| --- | --- | --- |
| Men, SSLDLD1 | $LP=-7.5153+0.0187\times age-0.6199\times Race_{black}-0.3336\times Race_{other}+0.0847\times BMI+0.9901\times AST\_G+0.0760\times\beta carotene\_G+0.5824\times GGT\_G+0.2792\times FPG\_G+0.9696\times insulin\_G+0.7959\times MCHC\_G$ | 0.7449 |
| Men, SSLDLD2 | $LP=-7.9107+6.8752\times WHR-0.2329\times Race_{black}-0.4285\times Race_{other}+0.6518\times Cpeptide\_G$ | 0.7039 |
| Women aged <55 years, SSLDLD1 | $LP=-6.5842+0.0190\times age+1.0779\times ALT\_G+1.1425\times\beta carotene\_G+0.3521\times HbA1c\_G+0.6356\times TG\_G+1.5319\times insulin\_G$ | 0.6598 |
| Women aged <55 years, SSLDLD2 | $LP=-5.7153+3.1907\times WHR+0.9615\times ASTALTratio\_G+0.4207\times FPG\_G+0.3966\times Cpeptide\_G$ | 0.6635 |
| Women aged ≥55 years, SSLDLD1 | $LP=7.7955+0.0865\times BMI-12.8884\times\beta carotene\_G+0.5397\times HDL\_G+1.3958\times insulin\_G$ | 0.7431 |
| Women aged ≥55 years, SSLDLD2 | $quation for {SSLDLD2}_{female\geq55}: -6.3677+4.2640\times WHR+0.4600\times ASTALTratio\_G+1.0028\times Cpeptide\_G$ | 0.6763 |

**TABLE S10. Coding definitions for predictors used in the SSLDLD models.**

| **Predictor term** | **Coding definition** |
| --- | --- |
| Race_black | 1 if Black; 0 otherwise |
| Race_other | 1 if race/ethnicity other than White or Black; 0 otherwise |
| AST_G | 2 if AST >37 U/L; 1 otherwise |
| ASTALTratio_G | 2 if AST/ALT ratio <1; 1 otherwise |
| βcarotene_G / BCP_G | 2 if β-carotene <2 μg/dL; 1 otherwise |
| GGT_G | 2 if GGT ≥51 U/L in men; 1 otherwise |
| FPG_G | 2 if fasting plasma glucose ≥100 mg/dL; 1 otherwise |
| HbA1c_G | 2 if HbA1c ≥5.7%; 1 otherwise |
| TG_G | 2 if triglycerides ≥150 mg/dL; 1 otherwise |
| HDL_G | 2 if HDL <50 mg/dL in women; 1 otherwise |
| insulin_G | 2 if insulin ≥20 μU/mL; 1 otherwise |
| Cpeptide_G | 2 if C-peptide ≥0.72 pmol/mL; 1 otherwise |
| MCHC_G | 2 if MCHC ≥37 g/dL; 1 otherwise |
| BMI | Continuous body mass index (kg) |
| WHR | Continuous waist-to-hip ratio (cm) |

Coding rules for predictors used in the SSLDLD models. Categorized predictors were generally encoded as 2 for the higher-risk category and 1 for the reference category, whereas BMI and WHR were retained as continuous predictors.

**SUPPLEMENTARY FIGURE LEGENDS**

**FIGURE S1. Nomograms for men.**(A) SLDLD1. (B) SLDLD2.

**FIGURE S2. Nomograms for women aged <55 years.**(A) SLDLD1. (B) SLDLD2.

**FIGURE S3. Nomograms for women aged ≥55 years.**(A) SLDLD1. (B) SLDLD2.

**FIGURE S4. Decision curve analysis comparing SLDLD1 with existing indices across the three NHANES III derivation strata.**(A) Men. (B) Women aged <55 years. (C) Women aged ≥55 years. Net benefit was plotted across threshold probabilities from 0.05 to 0.50 for SLDLD1, FLI, HSI, NAFLD-LFS, and US-FLI. The dashed line represents the treat-all strategy, and the dotted horizontal line represents the treat-none strategy. Across all three strata, SLDLD1 generally showed the highest or near-highest net benefit over most clinically relevant threshold probabilities, with the clearest advantage observed in men.
