# Supplementary material for: Development and Validation of the Steatotic Liver Disease Lab Data Index: A Refined Diagnostic Tool
Source: Can J Gastroenterol Hepatol. 2026 Jun 30;2026:7366206. doi: 10.1155/cjgh/7366206 (PMC13316952; doi:10.1155/cjgh/7366206)
Supplement: Supplementary file 1 — Supporting Information TABLE S1. Variables screened across the nine feature domains in the EWAS. TABLE S2. Predictor composition for SLDLD and SSLDLD indices across sex and age strata. TABLE S3. Paired DeLong tests comparing AUROC of SLDLD1 versus established steatosis indices. TABLE S4. Bootstrap internal validation of the stratified SLDLD1 models in the NHANES III derivation cohort. TABLE S5. Predictor selection frequencies of SLDLD1 across 1000 bootstrap resamples in the NHANES III derivation cohort. TABLE S6. Comparison of a pooled nonstratified SLDLD1 model and the stratified SLDLD1 framework in the NHANES III derivation cohort. TABLE S7. Impact of feature reduction on AUROC of SLDLD1 and SSLDLD1 in the NHANES III derivation cohort. TABLE S8. Portability assessment of SSLDLD1 after restricting predictors to variables available in the MJ Health Database. TABLE S9. Full equations and predictor composition for SSLDLD models across sex and age strata. TABLE S10. Coding definitions for predictors used in the SSLDLD models. FIGURE S1. Nomograms for men. (A) SLDLD1. (B) SLDLD2. FIGURE S2. Nomograms for women aged < 55 years. (A) SLDLD1. (B) SLDLD2. FIGURE S3. Nomograms for women aged ≥ 55 years. (A) SLDLD1. (B) SLDLD2. FIGURE S4. Decision curve analysis comparing SLDLD1 with existing indices across the three NHANES III derivation strata. [file CJGH-2026-7366206-s001.zip › Figure_S2_Nomograms_Women_U55_editable_panels.pptx]

## Slide 1
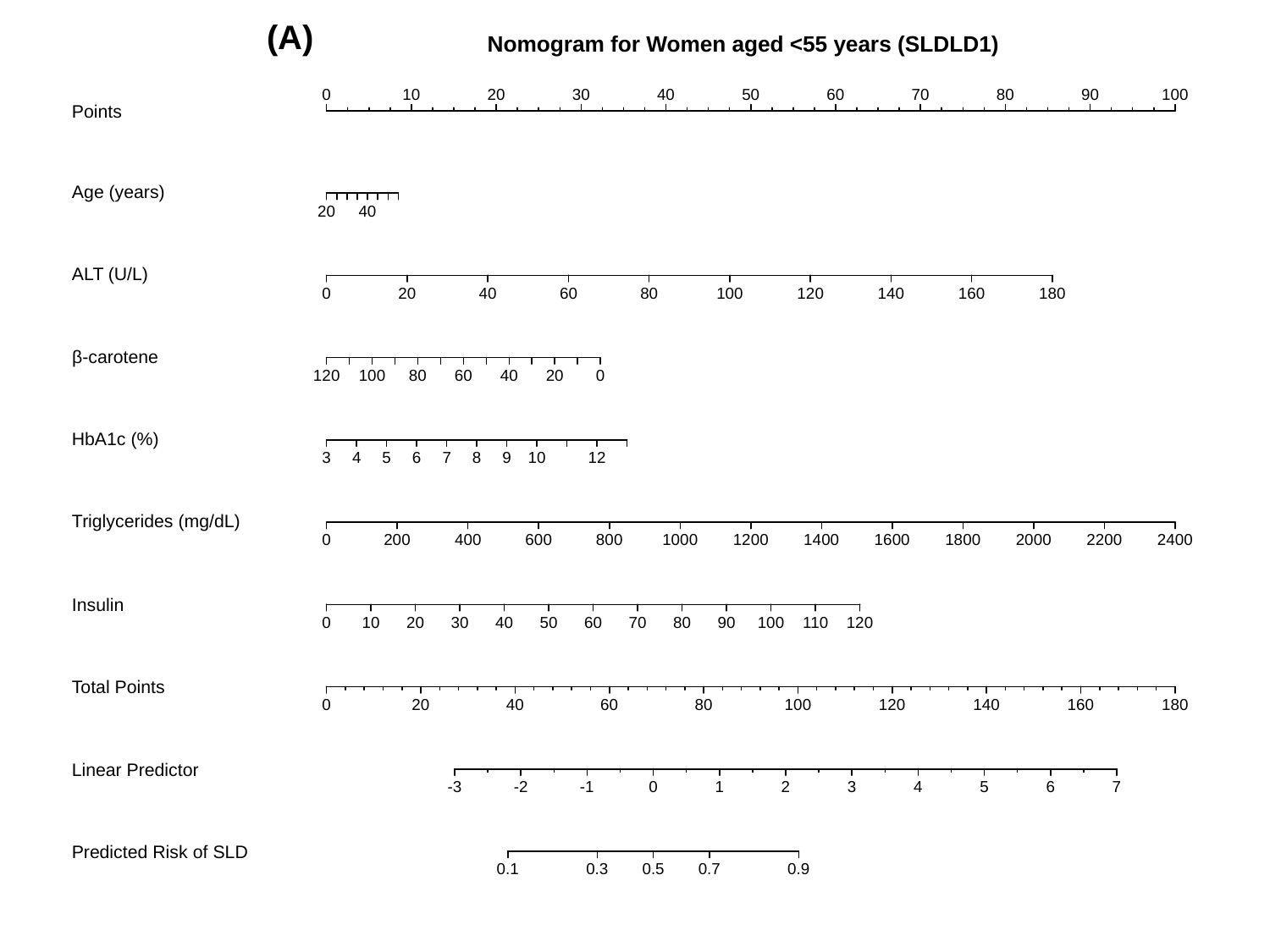

(A)
Nomogram for Women aged <55 years (SLDLD1)
0
10
20
30
40
50
60
70
80
90
100
Points
Age (years)
20
40
ALT (U/L)
0
20
40
60
80
100
120
140
160
180
β-carotene
120
100
80
60
40
20
0
HbA1c (%)
3
4
5
6
7
8
9
10
12
Triglycerides (mg/dL)
0
200
400
600
800
1000
1200
1400
1600
1800
2000
2200
2400
Insulin
0
10
20
30
40
50
60
70
80
90
100
110
120
Total Points
0
20
40
60
80
100
120
140
160
180
Linear Predictor
-3
-2
-1
0
1
2
3
4
5
6
7
Predicted Risk of SLD
0.1
0.3
0.5
0.7
0.9

## Slide 2
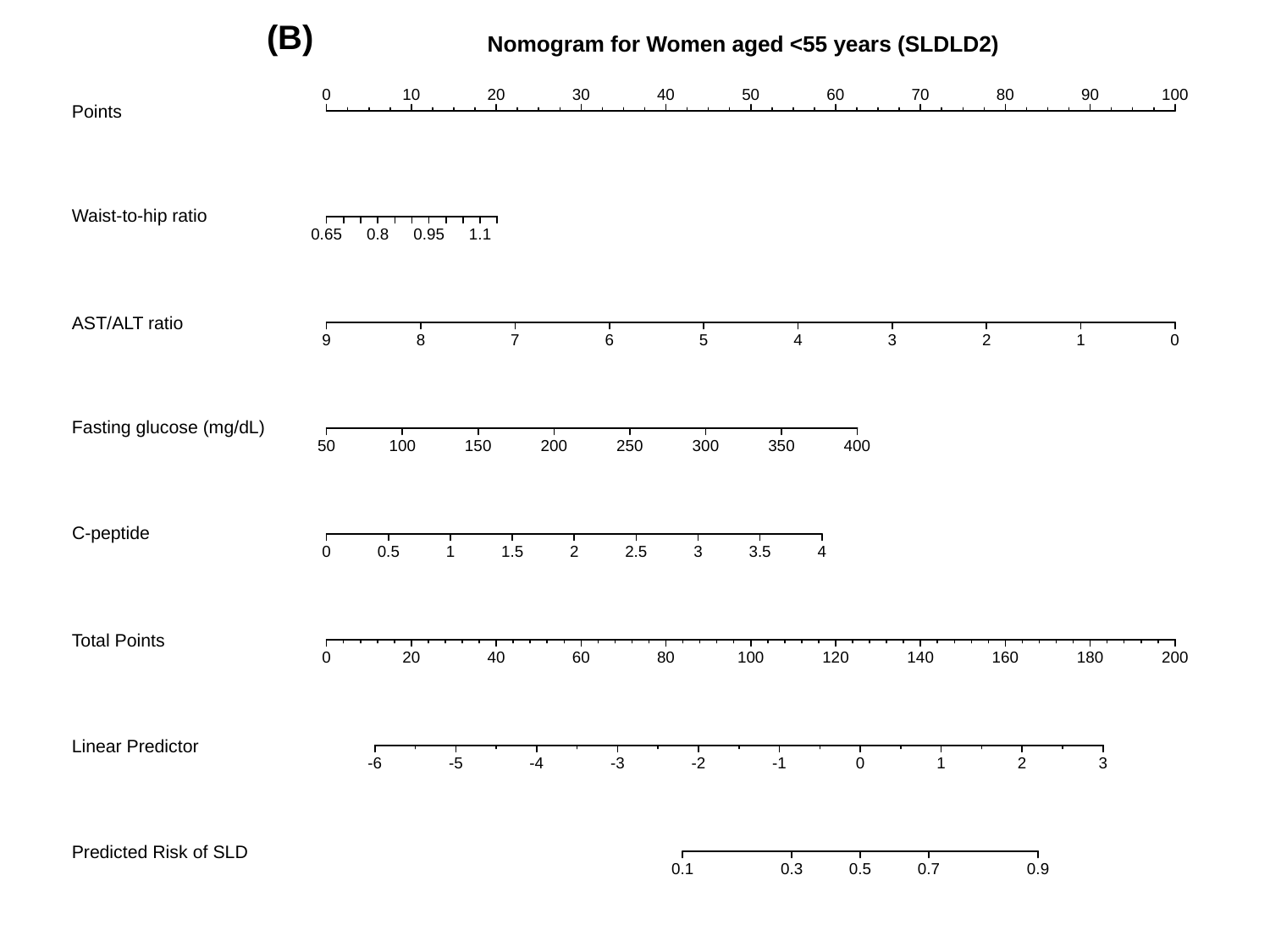

(B)
Nomogram for Women aged <55 years (SLDLD2)
0
10
20
30
40
50
60
70
80
90
100
Points
Waist-to-hip ratio
0.65
0.8
0.95
1.1
AST/ALT ratio
9
8
7
6
5
4
3
2
1
0
Fasting glucose (mg/dL)
50
100
150
200
250
300
350
400
C-peptide
0
0.5
1
1.5
2
2.5
3
3.5
4
Total Points
0
20
40
60
80
100
120
140
160
180
200
Linear Predictor
-6
-5
-4
-3
-2
-1
0
1
2
3
Predicted Risk of SLD
0.1
0.3
0.5
0.7
0.9
